# Positive sexuality: HIV disclosure, gender, violence and the law—A qualitative study

**DOI:** 10.1371/journal.pone.0202776

**Published:** 2018-08-24

**Authors:** Andrea Krüsi, Flo Ranville, Lulu Gurney, Tara Lyons, Jean Shoveller, Kate Shannon

**Affiliations:** 1 Gender and Sexual Health Initiative, Vancouver, British Columbia, Canada; 2 Department of Medicine, University of British Columbia, Vancouver, British Columbia, Canada; 3 Kwantlen Polytechnic University, Surrey, British Columbia, Canada; 4 School of Population and Public Health, University of British Columbia, Vancouver, British Columbia, Canada; Brown University, UNITED STATES

## Abstract

While a growing body of research points to the shortcomings of the criminal law in governing HIV transmission, there is limited understanding of how cis and trans women living with HIV (WLWH) negotiate their sexuality and HIV disclosure in a criminalized environment. Given the ongoing criminalization of HIV non-disclosure and prevalence of gender-based violence, there is a critical need to better understand the dynamics of negotiating sexual relationships and HIV disclosure among WLWH. We conducted 64 qualitative interviews with cis and trans WLWH in Vancouver, Canada between 2015 and 2017. The interviews were conducted by three experienced researchers, including a cis and a trans WLWH using a semi-structured interview guide. Drawing on a feminist analytical framework and concepts of structural violence, the analysis sought to characterize the negotiation of sexual relationships and HIV disclosure among WLWH in a criminalized setting. For many participants their HIV diagnosis initially symbolized the end of their sexuality due to fear of rejection and potential legal consequences. WLWH recounted that disclosing their HIV status shifted the power dynamics in sexual relationships and many feared rejection, violence, and being outed as living with HIV. Participants’ narratives also highlighted that male condom refusal was common and WLWH were not only subjected to the gendered interpersonal violence of male condom refusal but also to the structural violence of legislation that requires condom use but fails to account for the gendered power imbalance that shapes condom negotiation. Despite frequently being represented as a law that ‘protects’ women, our findings indicate that the criminalization of HIV non-disclosure constitutes a form of gendered structural violence that exacerbates risk for interpersonal violence among WLWH. In line with recommendations by, the WHO and UNAIDS these findings demonstrate the negative impacts of regulating HIV prevention through the use of criminal law for WLWH.

## Introduction

HIV is increasingly described as a chronic condition, thanks to the advent of modern HIV treatment technologies [1, 2]. People living with HIV, who have access to HIV treatment, can now live healthier lives and have a nearly unchanged life expectancy after diagnosis [3]. These advances also translate into negligible risk of HIV transmission in the context of sustained viral suppression [3, 4]. To promote medication uptake, and in an effort to reduce enduring HIV related stigma, activists and healthcare providers have largely been supportive of the discursive normalization of HIV as a chronic disease “like any other” (p.263) [5]. However, biomedical advances alone have been unable to depoliticize or desocialize HIV [5, 6]. Numerous intersecting social and structural vulnerabilities (e.g., poverty, housing instability, enduring HIV related stigma, racism, experiences of violence and gender inequality) undermine the ability of people living with HIV to reach this proclaimed ‘normalcy’ [1, 2, 5, 7, 8].

### Criminalization of HIV non-disclosure as structural violence

The criminalization of HIV non-disclosure profoundly disrupts the biomedical promise of living a ‘normal’ life with HIV. Despite strong recommendations by the World Health Organization (WHO) and the Joint United Nations Programme on HIV/AIDS (UNAIDS) against the use of criminal laws to govern HIV transmission, more laws are passed and more people than ever are prosecuted globally [9]. Criminalization, alongside continued perpetuation of stigma, contradicts constructions of HIV as “like any other” chronic condition. The concepts of structural violence/vulnerability and stigma have been previously used to frame how HIV is shaped by sociocultural, economic and political inequalities, including the exercise of power [6, 10, 11]. Structural violence is distinct from direct violence, in that it is embedded in social structures and draws attention to how “unequal power” shapes “unequal life chances” [12]. As such, the concept of structural violence here gives focus to how the criminalisation of HIV non-disclosure and gender inequality at the structural level shape experiences of intimacy, sexual relationships and disclosure among cis and trans women living with HIV (WLWH).

Canada has an assertive approach to criminalizing HIV non-disclosure and has among the highest number of convictions globally [13]. Using a condom or having a low or undetectable viral load is not sufficient in itself to exclude criminal liability for not revealing one’s HIV status to a sexual partner [14]. While there is no specific law in the *Canadian Criminal Code* to regulate HIV, HIV non-disclosure is most commonly prosecuted as aggravated sexual assault. Therefore, the legal test centers around whether a person would have consented to sex had they been aware of their partner’s HIV status. Sexual assault constitutes among the most severe charges in the *Canadian Criminal Code*. Of the 184 people who faced criminal changes related to HIV non-disclosure in Canada between 1989 and 2016, 33% were White, 23% Black, 6% Indigenous and 18% were known to have come to Canada as immigrants or refugees. Women make up 10% of all people charged with HIV non-disclosure. To date, 42% of women charged were Indigenous, highlighting the blatant overcriminalization of Indigenous women and WLWH in Canada [15]. In a positive development announced by the Canadian Government in December 2017, a Department of Justice report recommends a more limited application of the criminal law to HIV non-disclosure cases. The report explicitly recognizes HIV as a public health matter and recommends the Attorneys General at the Provincial level lay criminal charges in HIV non-disclosure cases only as a last resort [16].

### The complexities of HIV disclosure

There is now a significant body of public health as well as social science literature examining the complex dynamics of HIV disclosure [5, 17, 18]. HIV disclosure has been associated with some potential benefits including, improved access and adherence to treatment [19], increased social support, reduced stigma [20] and, in the context of sexual relationships disclosure, has been linked to reduced transmission risk behaviours [21].

However, despite potential benefits, HIV disclosure is a complex process [22]. Commonly, HIV disclosure is not a one time or ‘all or nothing’ event, as the law may conceptualize it. Indeed, disclosure is highly contingent upon social relations and locations. Thus, the decision and timing of disclosure differs depending on the social context and the nature of a relationship and also whether stigma, violence or loss of social status are expected [22, 23]. In sexual relationships, disclosure may be less common with casual partners, or in the context of sex work, especially when a condom is used or a person’s viral load is undetectable [23].

Given pervasive gender inequality [17, 18], WLWH face numerous barriers and risks related to HIV disclosure, including fear of violence, abandonment, stigma, or loss of economic stability [17, 24, 25]. Fears of HIV disclosure should be contextualized with the elevated prevalence of gender-based violence WLWH experience, that is often linked to their HIV status [26]. Estimates suggest that 68 to 95 percent of WLWH experience intimate partner or sexual violence in their lifetime [26–29], with transgender WLWH experiencing a particularly elevated burden of lifetime sexual violence [30]. Likewise, social and economic marginalization or dependence, such as low-wage employment and co-habitation, have been identified to reduce HIV disclosure to partners [31].

In Canada, and other environments where HIV non-disclosure to sexual partners is criminalized, the fears and apprehensions that shape disclosure among WLWH are augmented by the threat of criminal charges, public exposure and incarceration [32–34]. Existing social science and epidemiological research on the criminalization of HIV non-disclosure has focused primarily on the experiences and understandings of people living with HIV/AIDS regarding the criminal laws related to HIV and the relationship between such laws and sexual risk behaviours [35–38]. Indeed, the application of the criminal law disproportionately impacts marginalized people living with HIV/AIDS [38], and criminalization of HIV non-disclosure can exacerbate HIV-related stigma [39], interfere with access to HIV testing [40], and complicate relationships between health care providers and people living with HIV/AIDS [41]. However, much less is known about the gendered impact of the criminalization of HIV on WLWH. Therefore, the focus of this analysis is on how cis and trans WLWH negotiate sexual relationships and HIV disclosure in a criminalized context.

## Methods

This study draws from semi-structured interviews conducted with 64 participants in the context of the SHAWNA project. SHAWNA (*Sexual Health and HIV/AIDS*: *Women’s Longitudinal Needs Assessment)* is a participatory community-based longitudinal research project focused on examining the social, policy, legal, gender and geographic gaps in cis and trans women’s sexual health and HIV care across Metro Vancouver, Canada. The project was initiated after extensive community consultation with WLWH, HIV service providers, community organizations and clinicians and follows meaningful involvement of women living with HIV/AIDS (MIWA) and Meaningful involvement of people living with HIV/AIDS (MIPA) principles [42]. The research team, including peer research associates, nurses, and interviewers/ outreach workers, includes people with diverse backgrounds and experiences, including WLWH across various roles in the project.

The qualitative interview guide was developed collaboratively with WLWH and HIV service providers. It is designed to allow exploration of a broad range of social and structural factors shaping participants’ lives, including issues related to healthcare and social service access, sexual health, and HIV related stigma. The primary topic areas included in the interview guide were: health & wellness; HIV treatment experiences; sexual and reproductive health; parenting; HIV-related stigma; housing and if applicable incarceration.

Participants from the SHAWNA cohort study were invited to participate in qualitative interviews during their bi-annual cohort study visits at one of two research offices in Vancouver and through peer outreach to HIV support services and advocacy organizations in Metro Vancouver. Prospective participants were told that the study aims to better understand the experiences of cis and trans WLWH in accessing health and social supports to identify barriers to optimal health and wellbeing. Eligibility included identifying as a cis or trans women living with HIV, and residing or accessing HIV support services in Metro Vancouver. Sampling aimed to reflect variation in demographics such as ethnicity, age, and gender, to ensure the representation of Indigenous, Black/African and trans WLWH. While, the composition of our sample was intended to reflect the diversity of WLWH in Metro Vancouver, it is not intended to be ‘representative’.

Three experienced interviewers, including one cis and one trans WLWH, conducted the qualitative interviews between July 31^st^, 2015 and February 16^th^, 2017. All participants provided informed written consent. Interviews lasted between 60 and 120 minutes. Participants were remunerated with a CAD $30 honorarium for their time, expertise and travel. The study holds approval by the Providence Healthcare/University of British of Columbia Research Ethics Board—H14-01073.

### Data Analysis

All audio recordings were transcribed verbatim and checked for accuracy. Data collection and analysis occurred concurrently. The research team discussed the content of interviews, emerging themes and coding framework throughout the data collection and analytic processes. The initial coding framework was based on key themes reflected in the interview guide, participants’ accounts and fieldnotes. Two Master level trained team members broadly coded interview transcripts, using ATLAS.ti. To establish inter-coder reliability, both team members coded the same transcripts independently and compared code application continually. Once inter-coder reliability was established, they conferred regularly to compare codes. In an iterative process, to advance beyond thematic description, the lead author fine coded the data relating to HIV disclosure to sex partners by drawing on socio-ecological and feminist analytic frameworks, including structural violence and vulnerability [43] to elucidate how WLWH negotiate HIV disclosure, intimacy and sexual relationships in a context where HIV non-disclosure is criminalized. Analysis also drew on the concept of intersectionality to elucidate the complex interrelationships between multiple social positionings such as gendered subordination, racialization, and poverty [44]. Pseudonyms are used to protect the confidentiality of participants.

## Results

### Participant characteristics

Participants included 64 WLWH, of which 58 were recruited from the SHAWNA cohort and thus had their HIV status confirmed through voluntary CD4 and viral load testing in the context of their clinical cohort study visit. The remaining six participants were recruited through HIV support and advocacy services and their HIV status was determined through self-report. Fifty-four participants were cisgender women, and 10 participants were transgender, defined as an individual whose gender identity differs from the sex assigned to them at birth. Of those 10, 3 identified as ‘transgender,’ 3 as ‘two spirit’, 3 as ‘women’, 1 as ‘gender queer’. The median age of participants was 46, with a range from 24 to 68. Thirty-one participants (48%) were Indigenous (inclusive of Inuit, Métis, and First Nations), nineteen participants (30%) identified as White, nine participants (14%) identified as Black/African and were predominantly new immigrants and refuges from various African countries. Five participants identified as mixed ethnicity or other visible minority (including Middle Eastern and Pacific Islander). See Table 1.

**Table 1:** Socio-demographic characteristics of study sample.

	Cis WLWH n = 54	Trans WLWH n = 10	Total n = 64
**Median Age**	46	48	46
**Ethnicity**			
Indigenous	25	6	31
White	17	2	19
Black/African	9	0	9
Mixed/other visible minority (including Middle Eastern, Pacific Islander)	3	2	5

### ‘My sexuality is broken’ HIV diagnosis as the end of sexuality

For many cis and trans women living with HIV (WLWH), despite the promise of living a ‘normal’ life with HIV, their diagnosis symbolized the end of sexual relationships due to fear of disclosure, fear of transmitting HIV to partners and fear of legal consequences. While for some participants their sexuality was complicated by past sexual trauma or menopause, HIV was a key factor that shaped women’s sexuality and in particular lack of sexual relationships. Many of the participants in our study mourned the end of their sexual relationships after diagnosis.

*I quit having sex when I was diagnosed and I’m telling you it was one of the hardest things in my life to do, because I was a very physical person*.

Amy, Cis, Indigenous

*[When I was diagnosed] I was all by myself […] I was afraid to have sex with anybody so I didn’t have a relationship. […] I couldn’t have a relationship cause I had to tell that person but other than that I live my life like I don’t have HIV*.

Susan Cis White

Living with HIV significantly complicated WLWH’s sexual relationships and was an important consideration in many WLWH’s decision to not engage in sexual relationships anymore. Indeed, for Susan, the imperative of ‘having to tell’ and therefore not having sex represented a key factor in the way her life differed from life before her diagnosis. Also, despite the recent medical advances that significantly reduce the risks of HIV transmission and the pledge that HIV represents a disease ‘like any other’, an intense fear of inadvertent HIV transmission to others weaved through many participants’ narratives. These narratives were closely linked to the persistent stigmatization of HIV and the rejection many women experienced after disclosing their HIV status to potential sexual partners, friends and family.

[Living with HIV] *has been a barrier for relationships*, *and friendships as well*. *You know I’ve had people*, *quit calling me*. *[…] I would tell them I’m HIV positive*. *Oh don’t worry that won’t affect our relationship*. *And then that’s the last time I hear of them*. *So*, *I scare people still*. *That fear’s still there*.

Violetta, Cis, Indigenous

A two-spirit participant, Taylor, who is not currently sexually active, also discussed how for them HIV diagnosis further complicated their sexuality and fears around it.

*I didn’t know about, my gay, my sexuality until later on in life. But when I [was diagnosed with] HIV, I was worried about that again. I was like a little kid again*.

Taylor, Two-spirit, Indigenous

Thus, Taylor’s narrative shows how for WLWH of gender and sexual minorities HIV criminalization can intensify stigma and fear around their gender and sexual identity.

### Fear of HIV disclosure pushes women to stay in abusive relationships

A small number of participants recounted better than expected experiences of HIV disclosure to sex partners. Despite intense apprehension to disclose her HIV status Heather recounted a positive experience:

*It was really hard to tell my partner but he supports me no matter what. [He] loves me unconditionally, accepts me for who I am. So that was pretty freaking awesome. Because it’s hard to get into a relationship when I’m carrying this [HIV] on me and then having to tell somebody*.

Heather, Cis, Indigenous

Fear of HIV disclosure, criminalization, rejection and loneliness can push women to stay in or return to abusive relationships, as highlighted in Yvonne’s narrative:

[I was in an] *abusive*, *controlling relationship*. *Just not a good thing*. *[I went back to him] Because I didn’t have to disclose or nothing*. *We could just do it*. *I don’t have to think that way when I’m with him*. *[…] It’s the wrongest reasons to stay in a relationship*. *You’re thinking it’ll change*, *it’ll change but it never changes*. *I really learned that men don’t change*, *women do the changing*. *[…] Men take advantage if you’re a woman with disabilities*. *They think we’re vulnerable*.

Yvonne, Cis, White/Asian

Yvonne speaks to the relief of not having to disclose her status to her abusive ex-partner and to an imbalance of power that marked her relationship, which can make WLWH susceptible to violence and abuse, and remain in abusive relationships. This is amplified in the current legal context, where threats of disclosure by an abusive partner can further reinforce power imbalances in abusive relationships. Yvonne also links the criminalization of HIV non-disclosure to other forms of criminalization, such as the criminalization of poverty and drug use. She uses cannabis (which on 1^st^ October 2018 will be legalized in Canada) to manage the side-effects of antiretroviral therapy and grows it on a small scale to support herself financially and supplement her modest disability pension.

*I feel like we’re criminals, and on a health issue. That’s wrong*. *We’re criminalized for our choice of medicine, as in cannabis. I’m criminalized for being in poverty because I’m ill; and criminalized because I’m HIV. That’s three criminalizations. What kind of country is this?*

Yvonne Cis, White/Asian

Yvonne’s narrative highlights how the criminalization of HIV intersects with other forms of criminalization WLWH experience.

### Violence and disclosure

Caroline, a woman in her early fifties who was diagnosed with HIV almost a decade ago, recounted how, after her diagnosis, she feared violence when faced with having to disclose her HIV status to her abusive husband, who she is now separated from. In an attempt to reduce the potential of a violent outburst by her partner, she asked a nurse for help in disclosing her HIV status to her partner.

*And I did not know how to break it to my husband because he was very abusive. And we had a health nurse show up. […] I got her to tell my husband cause I would’ve just been beaten up*.

Caroline, Cis, White

The burden of her new HIV diagnosis and the legal obligation to disclose her HIV status to her partner or fear criminal prosecution weighed so heavy on Caroline that it drove Caroline to the brink of suicide at that time. Caroline’s narrative highlights how the legal obligation to disclose one’s HIV status, or use a condom and have an undetectable viral load before engaging in sex with an existing partner, can put WLWH at risk of violence. This points to the importance of providing supports to WLWH in disclosing their HIV status to mitigate the potential for intimate partner violence.

Vanessa’s narrative highlights the interpersonal and structural violence of a legal approach that criminalizes HIV non-disclosure in the context of extreme sexual violence. Vanessa is a young Indigenous trans woman who recounted a traumatic experience of rape—an incident that she did not report to police for fear of HIV non-disclosure charges.

*I didn’t report it*. *I couldn’t*. *[…] All of a sudden halfway through* [the rape], *he just asked “Are you positive*?*” I’m like*, *how do you answer that*? *[…] And it went so wrong that*, *I couldn’t even scream*. *And I kinda like*, *was hesitant and I kinda paced and then I just said well um*, *I’m undetectable and I kinda*, *lingered*, *I froze*.

Vanessa, Trans, Indigenous

Vanessa described how this experience of rape, and the fact that she could not report it for fear of legal repercussions, left her feeling silenced and powerless.

*Shit happens to us but no one’s speaking up. No one is and, I’m tired of being, pushed underneath some carpet because as a woman, regardless of being trans or not, I’m without power*.

Vanessa, 60 Trans, Indigenous

Vanessa explicitly referenced her gender identity as a trans woman and highlighted how being a trans woman contributed to her powerlessness in the face of sexual violence and a legal context that prevented her from seeking legal recourse. This further highlights how gendered power imbalances; stigma and the criminalization of HIV non-disclosure intersect to shape the experiences of violence among WLWH.

### Fear of disclosure to third parties

In addition to violence and abuse, many participants feared that after HIV disclosure, their partners might disclose their status without consent to others [26]. This fear became reality for Ayomide, a young African woman and newcomer to Canada. Ayomide, on the advice of a nurse, had not yet disclosed her HIV status to a new partner of a few weeks. As required by law, they had used condoms and her HIV viral load was undetectable. Yet, she described feeling frightened due to her partner’s threats of disclosing her status after he had discovered HIV medication in Ayomide’s apartment.

*Well he caught me with the medicine. […] He asked, I explained, but he got so annoyed. He threatens me every day*. *It’s not good. […] He abused me too much. […] So he wants to do something bad to me because I didn’t tell him. So I said, “Well we were using condoms” […] He told me that he was going to tell my friends [Ayomide’s roommates]. […] So I don’t sleep […] He said he will disclose the secret to my friends. Insulting me every day and night, “Prostitute—Are you a stripper? How did you get the sickness?”*

Ayomide Cis, African

Ayomide was able to negotiate condom use and her viral load was undetectable. Therefore, by law, she was not required to disclose her HIV status. However, the criminalization of HIV non-disclosure nonetheless exerted significant pressure on Ayomide by evoking fears about prosecution, child custody loss and eviction from her shared apartment.

*The nurse gave me almost half bag [of condoms]. You see, because she knows I’m Black, and maybe she don’t want me to get a partner here. I have to be very careful. She told me that*. *She told me that when you give it [HIV] to somebody they get you to go to jail. So that is in the back of my mind. I don’t have family here, beside my small child. I see myself in jail, what kind of a life I’m making for my child?*

Ayomide, Cis, African

Ayomide also referenced the colour of her skin, indicating that she interpreted the information the nurse provided regarding the legal context of HIV disclosure in Canada and the requirement for condom use, also in the context of racist stereotypes that position black African women as promiscuous, and dangerous [45, 46]. Also, Ayomide’s narrative reveals misinformation about the legal situation of HIV non-disclosure in Canada, where HIV transmission is not a necessary condition for prosecution. Misinformation and misconceptions about the legal context were common in many participants’ narratives and were particularly pronounced among new immigrant WLWH.

### Negotiate condoms to avoid disclosure ‘Just don’t come back and bite me’

Participants emphasized that the negotiation of male condom use was very difficult and put them at increased risk of prosecution. As highlighted by Rebecca’s and Tabitha’s narratives, negotiating condom use was often difficult for participants and, above and beyond verbal requests and physically providing the condom, women had limited power over whether a condom was used.

*I didn’t lie, I just didn’t bring it up. I omitted, the fact that I was positive and just tried to get them to use condoms, you know. As much as I could but…*.

Rebecca, Cis, White

*At first, I thought he was using it [the condom], but then, by the time we’re done I realize, “Oh no, we weren’t. Because, I gave you one, it’s still not”- of course he would open it, but then you can tell that he didn’t use it. So we didn’t. […] So…it’s just…I try, but, most men the idea of those things, kind of like ends up arguing, “So you don’t trust me? Are you saying I have something else?” they kind of make you feel… So you just end up going, “Okay”. Even though you know it’s not- I just go like, “Whatever, man. This is not my life. I tried. So when this backfires, just don’t come back and bite me*.

Tabitha, Cis, African

When a male partner refuses to use a condom, WLWH find themselves in a double bind where they either risk violence and rejection if they disclose their serostatus, or they break the law and make themselves vulnerable to prosecution. Accounts of condom refusal by their male partners were common in many WLWH’s narratives. This constitutes a form of gendered intimate partner violence where a partner imposes an unprotected sex act outside of the wishes of WLWH. In this sense, WLWH are not only subjected to the gendered interpersonal violence of male condom refusal but, at the more upstream level, also to the structural violence of legislation that is gender blind and does not account for the gendered power imbalance that shapes condom negotiation.

A number of participants shared stories of disclosing their HIV status; yet their partners nonetheless insisted on sex without a condom. While this scenario from a medical perspective in the context of an undetectable viral load poses a negligible risk of HIV transmission, for many women there still remained the potential for harm caused by the law criminalizing HIV. Many WLWH voiced concern regarding the potential of legal repercussions due to the risk of a partner denying that he had been aware of their HIV status. This is highlighted by Ruth’ narrative who made sure that the fact she had disclosed her status to her partner was documented in her medical file in case her partner might bring forward HIV non-disclosure allegations in the future.

*“He was like, ‘I’m so tired of using condoms all the time, I just want to have sex without it.’ And so it was really hard for me, but it was his decision to not use it. But we had gone to nurses and had it documented that he knew*.

Ruth, Cis, White

This narrative shows that WLWH like Ruth and Caroline (see above) are resourceful and resilient in mitigating the gendered structural violence that is exerted by the criminalization of HIV non-disclosure.

## Discussion

In summary, our findings demonstrate how the criminalization of HIV non-disclosure shaped the negotiation of sexual relationships and HIV disclosure among WLWH and constituted a key barrier to living with HIV as a chronic disease “like any other”. While the number of women charged with HIV non-disclosure in Canada is low [47], our findings elucidate the symbolic and productive dimension of this legal approach in shaping how WLWH negotiate their sexual relationships and disclosure. Despite frequently being represented as a law that ‘protects’ women, our findings elucidate how the criminalization of HIV non-disclosure constitutes a form of gendered structural violence. For many women, HIV diagnosis initially symbolized an end to their sexual relationships due to fear of rejection and prosecution. Women recounted that disclosing their HIV status shifted the power dynamics in their sexual relationships and many feared rejection and violence. The fear of criminal prosecution figured prominently in women’s minds and was closely connected with fears of public exposure of their HIV status, child custody loss and barriers of reporting sexual violence to police for fear of counter charges. Additionally, our findings highlight that the conditions under which WLWH are exempt from the legal obligation of disclosing their HIV status in Canada, namely having a low or undetectable HIV viral load AND using a condom, are distinctly gendered. Participants’ narratives highlighted the gendered power imbalance in negotiating male condom use in heterosexual relationships and draw attention to how WLWH are not only subjected to the gendered interpersonal sexual violence of condom refusal by their cis male partners, but also to the structural violence of legislation that takes no account of gendered power imbalances that shape male condom use. Trans women’s negotiation is further complicated by concerns around disclosure of gender identity and feelings of powerlessness in seeking legal recourse.

Conceptualizing the criminalization of HIV non-disclosure as a form of gendered structural violence allows us to better appreciate how this legal approach perpetuates HIV related stigma and gender-based violence at the structural level and how it intersects with various structural vulnerabilities WLWH face. A substantial body of literature documents that experiences of gender-based violence and HIV infection are closely intertwined [48–50] and intersect with various structural factors such as poverty, racialization, gender identity, unemployment, housing instability, sex work and substance use [26, 30, 51–54]. The pathways through which HIV and intimate partner violence among cis heterosexual partners are connected has been eloquently outlined by Dunkel and Decker (2013), and includes direct transmission of HIV by violent partners but also indirect pathways, where experiences of past violence reduce victims’ capacity to negotiate the terms of sexual relations. This puts women at risk for unwanted sex and reduces their capacity to negotiate sexual encounters, including situations where women are coerced or pressured not to use condoms [53, 55–57]. Our findings build upon this research and highlight that the criminalization of HIV non-disclosure renders WLWH, who are already disproportionately affected by gender-based violence, at additional risk of violence from intimate partners. This contributes to some WLWH staying in abusive relationships, or fear blackmail where partners may use women’s serostatus as a form of control and threaten criminal prosecution or involuntary public disclosure of their status. Previous findings indicate that involuntary HIV disclosure is widespread—affecting approximately half of WLWH in our setting and is linked to increased risk of experiencing HIV-related violence [26]. Our findings further highlight the distinctly gendered and intersecting consequences of involuntary HIV disclosure to third parties, including fear of eviction from shared accommodation or fear of losing custody of children in response to prosecution for HIV non-disclosure. These fears were particularly pronounced among the Black/African new immigrant WLWH given the exorbitant housing costs in the Metro Vancouver area and comparatively low social assistance payments, as well as the obligation of government funded refugees to pay back settlement loans. Child custody loss for WLWH who had been separated from their extended kinship networks due to migration was a particularly daunting potential consequence of prosecution due to HIV non-disclosure. As such, our findings elucidate how the criminalization of HIV non-disclosure intersects with other structural vulnerabilities such as migration status, racialization and poverty.

Our findings build upon previous work documenting the complexities of HIV disclosure [17, 18, 24, 25], and the gendered power dynamics of negotiating condom use, a condition in addition to a ‘low’ viral load, necessary to negate the legal obligation for HIV disclosure in Canada. While condom negotiation in heterosexual relationships is shaped by a myriad of influences, including the nature of the relationship, alcohol or substance use, and partner characteristics [58], these influences are framed by a broader context of gendered power imbalances [59]. Men have the direct ability to decide if a male condom is used, while women have to negotiate the use of a condom [60]. Our findings highlight that WLWH may be misled by their partners as to whether or not a condom was used at all or removed prematurely. This brings to bear a clear gender based difference in controlling the logistics of condom use and ultimately the legal obligation to disclose one’s HIV status.

Our findings further resonate with previous work that showed how the request for using a condom by women may be perceived to signify concurrent or past ‘promiscuous’ sexual behaviours or as a sign of mistrust [58, 59, 61]. Women with a history of gender-based violence in particular have been found to be less likely to negotiate condom use and more likely to expect negative consequences in response to negotiating male condom use [56, 58, 62]. Previous research has also indicated that gender identity plays an important role in condom negotiation among trans women. Condom negotiation among trans women has been identified as closely linked to power imbalances, stigma and a strong need for affirmation of their gender identity as women [63, 64]. Taken together with the high prevalence of gender-based violence among WLWH [26–28, 30], our findings highlight the barriers WLWH face in negotiating condom use in a criminalized environment. WLWH are not only subjected to the gendered sexual violence of male condom refusal but, at the more upstream level, also to the structural violence of legislation that does not account for the gendered power imbalance shaping condom negotiation. Our findings indicate that WLWH carry an amplified gendered burden in negotiating condom use in order to be exempt from the legal obligation to disclose their HIV status, and resonates with previous work that highlighted that, among people living with HIV with a history of illicit substance use, women faced a disproportionate legal burden to disclose their HIV status [38].

Our findings also highlight the symbolic power of this legal approach where WLWH may fear counter charges and are therefore reluctant to report sexual violence to authorities. This places WLWH outside the societal protections other citizens can take for granted. Also, WLWH fear prosecution, even after HIV disclosure when partners insist on condomless sex. While from a medical perspective this scenario, in the context of an undetectable viral load, poses a negligible risk of HIV transmission, our findings show that, for many WLWH, there remained the potential for harm caused by the law due to fear a partner may deny knowledge about their HIV status. Our findings highlight the potential role of healthcare providers in assisting WLWH with the documentation of HIV disclosure to sexual partners. However, this is contingent on the willingness of a partner to meet with the provider, which may not be feasible in the context of casual relationships.

Given the overrepresentation of Indigenous women in this study, it must be noted that the experiences of HIV, violence, and disclosure are also situated within a social and structural context of colonialism, and the racialization of Indigenous women [53, 65, 66]. The ongoing effects of colonialism and oppression of Indigenous peoples and cultures, including displacement from land and residential schools [67], are inseparable from the current health inequities, disproportionate burden of violence, and overrepresentation in the criminal justice system Indigenous women face [53, 68, 69]. As such, Indigenous WLWH may face increased negative consequences related to the criminalization of HIV non-disclosure due to intersecting structural vulnerabilities including racism, economic discrimination and gendered power inequalities [53, 70, 71].

This study has limitations. Findings from this study may not be generalizable to non-Canadian settings given the specificity of the Canadian HIV non-disclosure case law. To exercise extra caution, we did not ask direct questions about HIV disclosure to sexual partners during the qualitative interviews because, unlike in the US context, under Canadian law the researcher—participant relationship is not privileged and has been previously challenged when third parties issued subpoenas to order access to research records [72]. Participants shared all narratives about HIV disclosure presented in this paper in the context of questions about sexual and reproductive health, as well as experiences of HIV related stigma. There is the potential that more direct questions around personal HIV disclosure practices to sexual partners might have led participants to recount even more pronounced negative experiences around this law. Most of the research to date is drawn from data with cis women and further research is needed to better understand how the criminalization of HIV non-disclosure shapes the negotiation of sexuality among trans women and gender non-conforming people living with HIV.

In conclusion, our findings indicate that the current criminal justice approach to HIV non-disclosure is not well equipped in taking into consideration the gendered complexities surrounding HIV disclosure and how it intersects with gender-based violence and various structural factors, such as racism and the ongoing negative effects of colonialism. HIV disclosure should be framed as a social justice and public health issue rather than a criminal issue. Laws should focus on the protection of the rights of WLWH, promote equality, guarantee sexual and reproductive rights, and ensure access to essential services and privacy [23]. This study documents how the criminalization of HIV non-disclosure constitutes a form of gendered structural violence and presents a key barrier to HIV representing a chronic condition “like any other”. The recent call by the Federal Canadian Government to limit using a criminal justice approach to regulate HIV prevention is a step in the right direction. As the Canadian Provinces move toward the implementation of these recommendations (e.g., drafting of prosecutorial guidelines, law reform), we hope that the results of the current study will be taken into consideration. Ultimately our findings highlight that the criminal law is not well equipped to consider the gendered power dynamics of HIV disclosure and should not be used as a governing tool of HIV prevention.
